# Gingival bleeding and lower number of natural teeth are predictive factors of low muscle strength in obese adults: a cross-sectional study

**DOI:** 10.3389/froh.2026.1683238

**Published:** 2026-02-18

**Authors:** Karina Sarno Paes Alves Dias, Virgílio Santana-Júnior, Luciana Mara Barbosa Pereira, Hérika Maria Silveira Ruas, Felipe Oliveira Bittencourt, Gefter Thiago Batista Correa, Stênio Fernando Pimentel Duarte, Juciane Fagundes Durães Benitez, Renato Sobral Monteiro Junior, Sérgio Henrique Sousa Santos, Desirée Sant'Ana Haikal, Alfredo Maurício Batista de Paula

**Affiliations:** 1 Graduate Program in Health Sciences, Health Research Institute, State University of Montes Claros, Montes Claros, Brazil; 2 Departments of Dentistry and Physiotherapy, Faculdade Independente do Nordeste, Vitória da Conquista, Brazil; 3 Institute of Research and Extension (INPES), Vitória da Conquista, Brazil; 4 Graduate Program in Food and Health, Institute of Agricultural Sciences, Federal University of Minas Gerais, Montes Claros, Brazil

**Keywords:** multivariate modeling, anthropometry, bioelectrical impedance analysis, chronic metabolic disease, handgrip strength, inflammatory biomarkers, oral health status, preventive screening

## Abstract

**Introduction:**

Obesity is a systemic chronic disease associated with age-related skeletal muscle weakness, particularly in older adults. Chronic oral diseases share risk factors with critical non-communicable, chronic systemic diseases, including obesity. Poor oral health, increased fat mass, and muscle weakness are linked to adverse health outcomes and substantial economic burdens worldwide. This study developed predictive models of skeletal muscle strength (SMS) based on demographic characteristics, anthropometric and body composition measurements, blood biochemical examinations, and oral health parameters in obese adults.

**Methods:**

This cross-sectional study was conducted between 2022 and 2024 and included 122 Brazilian obese adults (mean age: 41.1 ± 12.8 years; 66.4% female) who received care in primary public health services. Standard methods were used to obtain appendicular and overall anthropometric measurements. Fat, non-fat, and skeletal muscle mass were assessed with bioelectrical impedance method. Handgrip strength was measured with a hydraulic handheld dynamometer. Low SMS was defined as sex-specific HGS test values fell below the 25th percentile. Blood counts and circulating biochemical variables were measured using enzymatic, chromatographic, and mass spectrometric methods. The oral health exams evaluated dental and periodontal status. Multiple linear regression analysis was employed to identify the associations between SMS and independent variables.

**Results:**

Low SMS was identified in 35.2% of obese adults. The predictive model yielded an adjusted R-squared of 0.607 and a root mean square error of 5.182. Reduced SMS included female sex (*β* = −8.438; *p* < 0.001), lower height (*β* = 0.230; *p* = 0.010), higher body weight (*β* = −0.087; *p* = 0.022), lower estimated muscle mass (*β* = 0.140; *p* = 0.002), gingival bleeding (*β* = −3.881; *p* < 0.001), and greater tooth loss (*β* = −0.440; *p* < 0.001).

**Conclusion:**

This study developed and validated a predictive model for low SMS in obese adults in a public healthcare setting, utilizing demographic characteristics, anthropometric measurements, body composition assessments, clinical parameters, blood biochemical examinations, and oral health parameters. Poor oral health, characterized by gingival bleeding and tooth loss findings, is associated with a higher risk of SMS in obese adults. Multiprofessional integrated care strategies may facilitate the early identification of muscle weakness in this population within public health care settings.

## 1 Introduction

Obesity is a multifactorial, chronic, systemic, metabolic disease defined by excessive body fat accumulation with consequent weight gain. The etiology of obesity involves complex interactions between endogenous factors, such as genetic and epigenetic disturbances, although these account for only a small proportion of cases, and exogenous determinants, including socioeconomic status, excessive energy intake, appetite, lack of physical activity, changes in gut microenvironment and microbiome, and deficiencies in healthcare strategies for early detection and intervention in obesity management of individuals with the metabolic disease determinants (1). Obesity is diagnosed when an individual has a body mass index (BMI) of 30 kg/m^2^ or higher, with BMI serving as the most widely accepted parameter to define the condition (2). Obesity increases the risk of systemic chronic diseases and is associated with all causes of mortality (3). Furthermore, obesity and its long-term outcomes impose a significant financial impact on the public health systems worldwide (4). In Brazil, the pooled prevalence of adult obesity varies by 17%–20% (5).

During the progression of obesity, some individuals develop muscle weakness, identified as low skeletal muscle strength (SMS). Individuals with low SMS are more likely to develop functional disabilities, which may compromise mobility, physical strength, posture, performance, and dynamic balance (6). Handgrip strength (HGS) is measured using a handheld hydraulic dynamometer, which provides an assessment of isometric strength and indicates an individual's overall muscle strength (7). Age, sex, anthropometric factors (height, body weight, hand size, arm circumference), and hand dominance influence the HGS measurement. The HGS is a reliable marker for various health outcomes (7). Studies have demonstrated that individuals with obesity exhibit a high prevalence of low HGS, particularly among older adults, women, and those with more severe BMI classification. Moreover, low HGS has been associated with numerous adverse outcomes in individuals with obesity (8, 9). The association between obesity and low SMS has been well established in studies involving older individuals, with the adult population frequently underestimated (10).

Dental caries, periodontal disease, and tooth loss are clinical indicators of poor oral health that share several exogenous risk factors with obesity, including a hypercaloric diet, inadequate hygiene habits, and unfavorable lifestyle and socioeconomic conditions (11, 12). Risk factors contributing to poor oral health and obesity frequently coexist within the same individuals or populations (13). Moreover, chronic systemic inflammation and oxidative stress play significant roles in the relationship between chronic oral diseases and obesity (14, 15).

Clinical prediction models estimate an individual's absolute probability or risk that a condition or disease is present or absent during the prediction (16). Early detection of low SMS in obese adults may facilitate the adoption of multidisciplinary, innovative strategies to prevent sarcopenic obesity. Public health care systems require reliable, cost-effective, and accessible assessment methods for screening skeletal muscle weakness in obese adults within primary and secondary healthcare settings.

This study developed and validated a predictive model for low SMS in obese adults in a public healthcare setting, utilizing demographic characteristics, anthropometric measurements, body composition assessments, clinical parameters, blood biochemical examinations, and oral health parameters.

## 2 Materials and methods

The Transparent Reporting of a Multivariable Prediction Model for Individual Prognosis or Diagnosis guidelines (17) recommendations were followed to ensure adequate reporting of this single-center, observational, cross-sectional study, conducted between April 2020 and December 2022, in Vitória da Conquista, Bahia State (northeastern Brazil).

### 2.1 Participants

**Figure 1:**
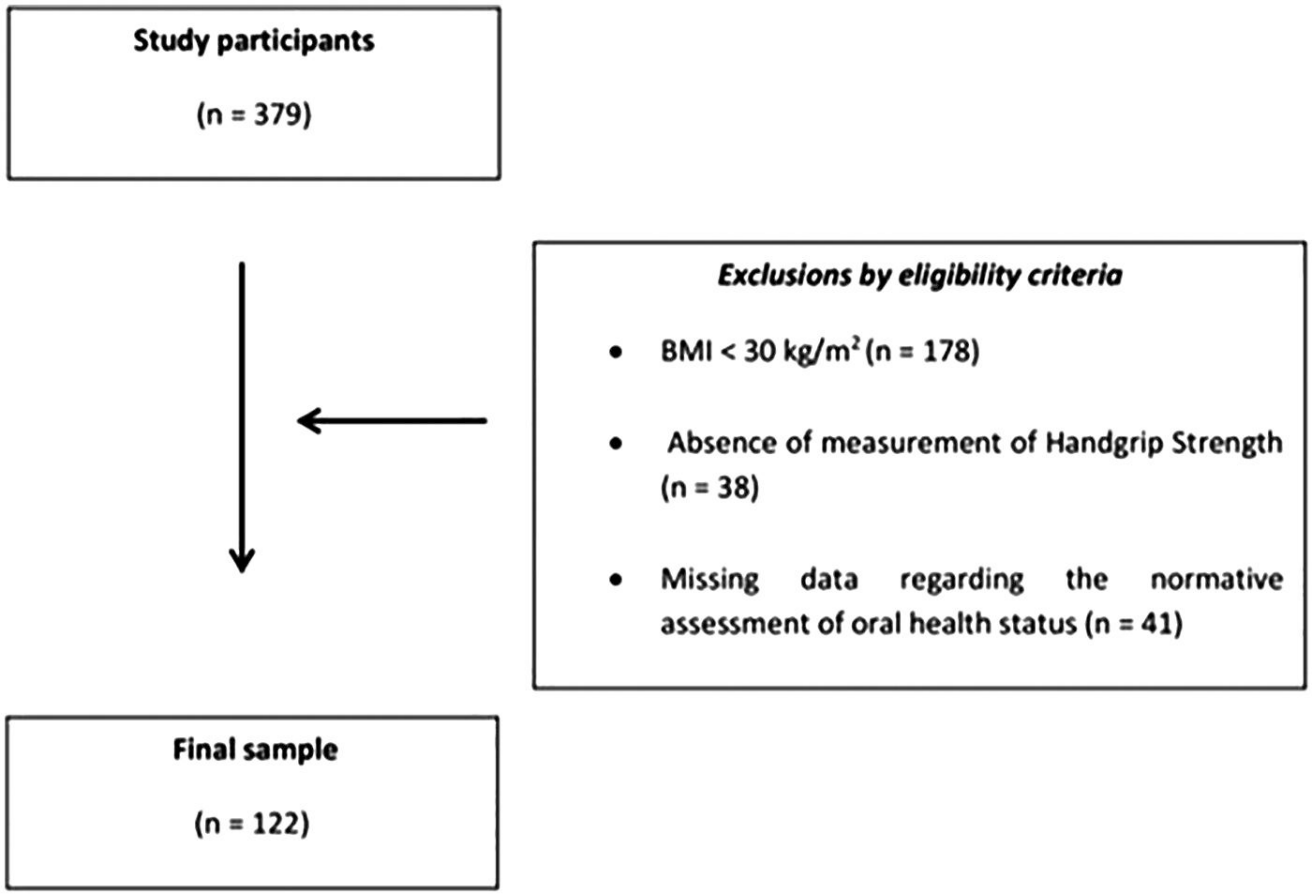
Flowchart of the selection process for participants in this study.

This study encompassed 122 adults diagnosed with obesity (mean age: 40.04 ± 14.13 years; 81 female and 41 male). G*Power 3.1 software (v. 3.1, HHU, Germany) was used to calculate the sample size, utilizing the *F*-test for multiple linear regression (fixed model, R^2^ ≠ 0), with a medium effect size (f^2^ = 0.15), eight predictive factors, and a significance level of 0.05. The *post hoc* analysis indicated a statistical power of 0.86. All participants were physically independent, with preserved cognitive and functional abilities, and regularly accessed primary health care services. Exclusion criteria included a diagnosis of an acute, local, or systemic inflammatory response, any untreated chronic cardiometabolic disease or comorbidities affecting the musculoskeletal system, use of medications influencing body composition, a history of orthopedic or locomotor diseases or sequelae, pain during walking, current bedridden status, a history of oncological treatment in the past five years, and a diagnosis of neurological disease. The flowchart of the sampling procedure is illustrated in Figure 1.

### 2.2 Predictive factors

#### 2.2.1 Tobacco smoking status and alcohol consumption

The tobacco smoking status was determined by self-report. Participants were categorized as “smokers” and “non-smokers”. Smokers were defined as current tobacco users who answered affirmatively to “Do you currently smoke tobacco? (yes, on most or all days or occasionally)”. Non-smokers were participants who responded never smoking or who had quit smoking for at least five years. Alcohol consumption status was also determined by self-report, classifying participants as “alcohol users” or “non-users”. Alcohol users reported current alcohol consumption (yes, on most or all days or occasionally), whereas non-users were those who never consumed alcohol or had abstained for at least one year.

#### 2.2.2 Anthropometric and body composition assessments

Anthropometric measurements were obtained using standardized and validated instruments (18). Height was measured using a fixed stadiometer (Cescorf, Brazil), and body weight was recorded to the nearest 0.1 kg with a calibrated balance beam scale (HBF222-T, Omron, Japan). The BMI was calculated as weight (kg) divided by the square of the height (m), with obesity classified as BMI ≥30 kg/m^2^ (19). Body composition was assessed by bioelectrical impedance analysis (BIA) via a multiple-frequency device (InBody120, InBody, South Korea) to estimate body water, fat-free mass, fat mass (FM), and %FM (FM/body weight). Resistance (R, Ohm) and reactance (Xc, Ohm) were measured at frequencies between 20 and 100 kHz. Two trained, blinded examiners performed the BIA method on all participants. The participant was lying supine on a stretcher with four members in contact with the electrodes. Body composition measurements using the BIA method were performed in all participants after an overnight fast, following standardized conditions: abstaining from alcohol for 12 h, emptying the urinary bladder at least 30 min before the test, refraining from physical exercise, and avoiding bathing before the test (20).

#### 2.2.3 Skeletal muscle mass, strength, and performance

Fat mass and skeletal muscle mass (SMM) were estimated using the BIA device. To calculate the SMM index, the SMM was adjusted by the height of each participant in this study (21). The maximum voluntary HGS was measured as an indicator of SMS using a hydraulic hand dynamometer (PC5030J1, Jamar, Brazil). Participants were instructed to exert as much pressure as possible on the device for at least 4 s. They performed three trials with each hand, separated by 20-second intervals. The highest value obtained from either hand represented the maximum muscle strength. Normal and low HGS cut-off values were established using reference values (<25th percentile) from a Brazilian adult population, stratified by sex and age group (18–29, 30–39, 40–49, and 50–59 years) (10).

#### 2.2.4 Hematological and biochemical parameters

Hematological analyses were conducted using the Vitros 5600 analyzer (Ortho Clinical Diagnostics, USA). The parameters measured were complete blood count: red blood cells, hemoglobin, hematocrit, mean corpuscular volume, mean corpuscular hemoglobin, mean corpuscular hemoglobin concentration, platelets, and white blood cells. Differential leukocyte count (neutrophils banded, neutrophils segmented, monocytes, eosinophils, and lymphocytes) was determined by evaluating Wright-Giemsa-stained blood smears and quantifying leukocyte percentages across ten fields. Serum samples were stored at 4 °C and analyzed within eight hours of collection. Hemolyzed or lipemic samples were excluded from the study. Biochemical tests were performed with the Vitros 5600 device (Ortho Clinical Diagnostics, USA). Assessed indices included triglycerides, total cholesterol, and fasting blood glucose. C-reactive protein (CRP) was examined using a modified Behring Latex-Enhanced assay on the analyzer system (Behring Nephelometer, Behring Diagnostics, USA). Quality control protocols encompassed both within- and between-assay procedures, and the coefficient of variation for the method ranged between 3.2%–16.1% over the data collection period. The assay detected CRP concentration of 0.22 mg/dl, and values below this threshold were considered undetectable. Eutrophic control participants (BMI >18.5 and <25 kg/m^2^) of the same sex and similar age served as the control group. Measurements adhered to the International Federation of Clinical Chemistry and Laboratory Medicine guidelines.

#### 2.2.5 Oral health status

The oral health was assessed through clinical examinations of the teeth and periodontal tissues by two trained dentists following the Oral Health Assessment criteria established by the World Health Organization (22). The decayed, missing, and filled teeth index was used to determine dental caries and the dental treatment needs of each participant. Additional dental assessments included quantifying the number of remaining natural teeth, decayed teeth, and missing teeth (NMT), where the remaining natural teeth accounted for erupted, permanent natural teeth. Dental biofilm was diagnosed as a sticky, colorless, or pale-yellow deposit on the tooth, which becomes visible upon accumulation and mineralization. Both dental caries and periodontal disease are biofilm-dependent diseases manifested within the oral environment. Periodontal assessment comprised evaluation of periodontal probing depth, the distance between the sulcus depth and the gingival margin, and bleeding on probing (where the internal surface of the gingival sulcus bled after 30 s touched with a probe), and clinical attachment level (the distance between the sulcus depth and the cementum-enamel junction of all existing natural teeth). The gingival index assessed bleeding at the free gingival margin after gentle probing, performed at six sites per tooth (mesiobuccal, buccal, distobuccal, distolingual/palatal, lingual/palatal, and mesiolingual/palatal). Gingivitis was diagnosed when bleeding on probing occurred at more than 10% of sites, without loss of attachment (23). Periodontitis was measurable in two or more non-adjacent teeth or ≥3 mm on free surfaces, with pocketing >3 mm at the exact location (24).

### 2.3 Ethical aspects

All research procedures conformed with the institutional and national ethical standards (CAAE no. 50251721.0.0000.5578, process number: 4.931.111), as well as the 1964 Helsinki Declaration and subsequent amendments. All participants signed a consent form informing them about the study's research design.

### 2.4 Predictive diagnostic model development and validation

Outliers were identified using boxplots and standardized residuals and removed prior to modeling. Multiple linear regression analysis was conducted with a bidirectional stepwise approach, adopting the following criteria: entry *p* < 0.20, retention *p* < 0.05, and absence of multicollinearity (variance inflation factor <10), and clinical plausibility. Predictive performance was evaluated using mean square error (MSE), root mean square error (RMSE), mean absolute error (MAE), and mean absolute percentage error (MAPE). RMSE was interpreted in the same unit as the dependent variable (kgf), and predictive error was classified as low when the RMSE represented less than 20% of the sample mean of handgrip strength. In this study, the mean HGS was 29.3 kgf; thus, RMSE = 5.162 kgf met this criterion. MSE was interpreted as the squared RMSE. Model comparison between M1 and M0 employed Akaike information criterion (AIC) and Bayesian information criterion (BIC). Internal validation was performed by randomly splitting the sample into a training set (*n* = 98) and a test set (*n* = 24). The Durbin–Watson test was used to verify independence of residuals.

### 2.5 Statistical analysis

Descriptive statistics were used to characterize the sample's demographic, anthropometric, body composition, SMM, SMS, clinical, serum biochemical, and oral health parameters. Numerical variables were presented as mean ± standard deviation and 25th, 50th, and 75th percentiles. Categorical variables were expressed as absolute and relative frequencies. The SMS served as the dependent variable. Spearman correlation analysis was performed for continuous independent variables. All analyses were conducted using SPSS (v. 25.0, IBM, USA).

## 3 Results

The mean and % findings of the demographic and anthropometric data, SMM, SMS, physical performance, and body composition parameters in obese adults are presented in Table 1. Most participants were women (66.4%, *n* = 81). The mean age of participants was 41.1 ± 12.8 years (women, 25.4 ± 5.2 years; men, 36.9 ± 7.8 years). The overall mean body weight and height were 97.4 ± 18.4 kg and 165.5 ± 9.2 cm, respectively (men, 109.3 ± 19.1 kg and 1.74 ± 0.08 m; women, 91.3 ± 14.7 kg and 1.60 ± 0.05 m). The mean BMI was 35.4 ± 5.0 kg/m^2^. Participants were categorized as class I (30–34.9 kg/m^2^; 57.4%), II (35–39.9 kg/m^2^; 25%), and III (≥40 kg/m^2^; 6%).

**Table 1:** Analysis of demographic and anthropometric data, habits body composition, muscle strength, and physical performance parameters of the participants (*n* = 122).

Parameters	*n* (%)	Mean ± SD	P25	P50	P75
Demographic					
Age (years)	–	41.1 ± 12.8	31.6	40.8	50.1
Sex					
Female	81 (66.4%)				
Male	41 (33.6%)				
Habits					
Smoking					
Yes	5 (12.3)	–	–	–	–
No	107 (87.7)	–	–	–	–
Alcohol consumption					
Yes	26 (21.3)	–	–	–	–
No	96 (78.7)	–	–	–	–
Anthropometric data					
Body weight (kg)		97.4 ± 18.4	84.4	91.7	106.2
Height (m)		165.5 ± 9.2	159.8	164.0	171.3
Body mass index (kg/m^2^)		35.4 ± 5.0	31.6	34.2	38.0
Obesity grade I	70 (57.4)	–	–	–	–
Obesity grade II	35 (28.7)	–	–	–	–
Obesity grade III	17 (13.9)				
Body composition (bioelectrical impedance analysis)					
Fat mass (kg)	–	42.2 ± 10.4	33.9	43.6	49.3
Skeletal muscle mass (kg)	–	32.3 ± 13.0	22.8	27.5	36.8
Skeletal muscle strength					
Handgrip strength (kgf)	–	29.3 ± 8.3	23.2	29.5	32.2
Blood cell count					
Red blood cells	–	4.8 ± 0.5	4.5	4.7	5.1
Hemoglobin (g/dl)	–	14.2 ± 1.6	13.3	14.0	15.2
Hematocrit	–	42.7 ± 3.8	40.0	42.0	46.0
Total leukocytes (cells/mm^3^)	–	7,515.8 ± 2,371.3	6,117.5	7,190.0	8,552.5
Blood glucose (mg/dl)	–	99.3 ± 32.4	85.8	92.0	103.0
Total cholesterol (mg/dl)	–	196.2 ± 41.9	161.0	194.5	226.8
Triglycerides (mg/dl)	–	255.4 ± 136.7	132.2	189.0	289.0
Albumin (g/dl)	–	4.4 ± 0.4	4.1	4.4	4.7
Inflammatory biomarker					
High sensitivity C-reactive (mg/dl)	–	0.76 ± 1.35	0.18	0.35	0.76
Oral health					
Oral hygiene					
Regular	114 (93.4)	–	–	–	–
Irregular	8 (6.6)	–	–	–	–
Use of dental floss					
Regular	51 (41.8)	–	–	–	–
Irregular	71 (58.2)	–	–	–	–
Number of decayed teeth	–	2.3 ± 2.8	0.0	1.0	3.0
Number of missing teeth	–	7.3 ± 5.4	4.0	6.0	10.0
Periodontal probing depth	–	3.8 ± 8.6	0.0	0.0	3.1
Gingival index	–	18.3 ± 18.2	4.6	12.6	27.1
Clinical attachment level	–	3.7 ± 2.7	2.0	4.0	5.0
Bleeding on probing					
Yes	52 (42.6)	–	–	–	–
No	70 (57.4)	–	–	–	–
Periodontal disease					
Gingivitis	46 (37.7)	–	–	–	–
Periodontitis	28 (23.0)	–	–	–	–

Data are presented as *n* (%), mean (± standard deviation, SD), and percentiles 25, 50, and 75. P, percentile.

The SMM was estimated using the BIA method. The mean SMM values were 36.9 ± 7.7 kg for men and 25.4 ± 5.2 kg for women (Table 1). The SMS of each participant was obtained by measuring the HGS. The mean HGS across all participants was 31.3 ± 9.9 kgf (range: 20.0–50.0 kgf). Most participants (64.8%) exhibited normal SMS. Low HGS was identified in 46.3% (*n* = 19) of male participants and 29.6% (*n* = 24) of female participants. Among adult women, the mean HGS was 25.4 ± 5.2 (range: 14.0–38.6 kgf); among adult men, the mean was 36.9 ± 7.8 (range: 20.0–50.3 kgf).

The measured hematological parameters among all participants included a mean red blood cell count of 4.8 million/mm^3^, hemoglobin of 14.2 g/dl, and hematocrit of 42.7%. The mean blood glucose concentration was 99.3 mg/dl. Mean serum total cholesterol was 196.2 mg/dl, and high triglyceride levels of 255.4 ± 136.7 mg/dl. The mean serum albumin level was 4.4 ± 0.4 g/dl. The mean high-sensitivity C-reactive protein was 0.76 ± 1.35 mg/dl, with 55.7% of participants at high cardiovascular risk and 23.8% showing acute inflammation (Table 1).

Most participants reported regular toothbrushing (93.4%). However, only 41.8% reported regular dental flossing. The mean NMT was 7.3 ± 5.4, and the mean number of decayed teeth was 2.3 ± 2.8. Active dental caries lesions were diagnosed in 67.8% of participants. Visible tooth biofilm was present in 76.2% of participants. Bleeding on probing occurred in 42.6% of participants, and periodontal disease was diagnosed in 60.7% of obese adults.

The results of the correlation analysis between SMS and independent variables are shown in Table 2. On one hand, SMS demonstrated a significant moderate positive correlation with height (*r* = 0.598; *p* < 0.001) and SMM (*r* = 0.442; *p* < 0.001). On the other hand, SMS was moderately negatively correlated with FM (*r* = −0.376; *p* < 0.001) and age (*r* = −0.316; *p* < 0.001). Among laboratory markers, SMS correlated positively with red blood cell count (*r* = 0.449; *p* < 0.001), hemoglobin (*r* = 0.503; *p* < 0.001), hematocrit (*r* = 0.519; *p* < 0.001), and albumin (*r* = 0.339; *p* < 0.001), with effect sizes ranging from moderate to high. The high-sensitivity C-reactive protein concentration correlated negatively and significantly with SMS (*r* = −0.199; *p* = 0.028). The remaining biochemical variables did not exhibit statistically significant correlations. Regarding the oral health status of participants, SMS was moderately negatively correlated with NMT (*r* = −0.487; *p* < 0.001) and number of decaying teeth (*r* = −0.222; *p* = 0.014), suggesting an association between poor oral health and reduced SMS in obese adults.

**Table 2:** Correlation analysis between skeletal muscle strength and demographic, anthropometric, clinical, and laboratory data, and oral health status in obese adults (*n* = 122).

Parameters	Skeletal muscle strength in obese adults		
	R	*p*	R^2^
Age (year)	−0.316	<0.001	0.099
Body weight (kg)	0.407	<0.001	0.165
Height (cm)	0.598	<0.001	0.357
Body mass index (kg/m^2^)	−0.019	0.832	0.000
Skeletal muscle mass (kg)	0.442	<0.001	0.195
Fat mass (kg)	−0.376	<0.001	0.141
Red blood cells (million cells/mm^3^)	0.449	<0.001	0.201
Hemoglobin (g/dl)	0.503	<0.001	0.253
Hematocrit (%)	0.519	<0.001	0.269
Total leukocytes (cells/mm^3^)	−0.036	0.694	0.001
Blood glucose (mg/dl)	−0.127	0.162	0.016
Total cholesterol (mg/dl)	0.151	0.097	0.022
Triglycerides (mg/dl)	0.138	0.131	0.019
Albumin (g/dl)	0.339	<0.001	0.114
High-sensitivity C-reactive protein (mg/dl)	−0.199	0.028	0.039
Missing teeth	−0.487	<0.001	0.237
Decayed teeth	−0.222	0.014	0.049

A multiple linear regression model was constructed for predictive analysis. Demographic characteristics, health habits, anthropometric measurements, clinical findings, hematological and blood biochemical examinations, and oral health parameters were evaluated as predictors of low SMS in obese adults. Variable selection was based on statistical significance (*p* < 0.05) and clinical relevance. The M1 was then compared to the M0. Additionally, to assess internal validity, the sample was randomly divided into training (*n* = 98) and test (*n* = 24) sets. After identifying and removing outliers, the M1 demonstrated superior performance than the M0, as summarized below (Table 3).

**Table 3:** Predictive diagnostic model validity of skeletal muscle weakness in obese adults (*n* = 122).

Model	Adjusted R^2^	RMSE	AIC	BIC	DW	*p*
M0	0.000	8.262	864.469	870.077	1.372	<0.001
M1	0.607	5.182	757.363	782.600	1.731	0.107

RMSEA, root means square error of approximation; AIC, akaike information criterion; BIC, Bayesian information criterion; DW, Durbin-Watson.

He M1 model accounted for 60.7% of SMS variability (adjusted R^2^ = 0.607). Predictive accuracy indices were MSE = 26.649, RMSE = 5.162, MAE = 4.181, and MAPE = 15.8%. Compared with M0, M1 presented lower AIC and BIC values. The Durbin-Watson statistic test was 1.731 (*p* = 0.107), indicating no residual autocorrelation. In the test set, the model explained 56% of variability in handgrip strength (R^2^ = 0.56). Analysis of the regression coefficients indicated statistically significant associations (*p* < 0.05) between low SMS and variables, including female sex, presence of blood on probing, higher NMT, lower height, increased body weight, and lower SMM were significantly associated with low SMS (*p* < 0.05). Tooth loss and gingival bleeding were negatively associated with SMS, suggesting that compromised oral health may affect SMS in obese adults. Height and SMM were positively associated with SMS. In contrast, body weight showed a negative association, potentially reflecting the adverse impact of FM on SMS (Table 4).

**Table 4:** Relationship between skeletal muscle weakness and independent variables analyzed via multiple linear regression (*n* = 122).

Parameters	Skeletal muscle weakness in obese adults		
	*β* coeficient	*t*	*p*
Intercept	−0.018	−0.001	0.999
Female	−8.438	−5.305	<0.001
Bleeding on probing	−3.881	−3.850	<0.001
Number of missing teeth	−0.440	−4.582	<0.001
Height (m)	0.230	2.616	0.010
Body weight (kg)	−0.087	−2.320	0.022
Fat mass (kg)	0.089	1.643	0.103
Skeletal muscle mass (kg)	0.140	3.225	0.002
Total cholesterol	0.010	0.811	0.419

## 4 Discussion

This study diagnosed a low SMS in 35.3% of obese adults. Furthermore, female sex, lower height, increased body weight, SMM estimative, gingival bleeding, and greater NMT significantly predicted low HGS. Dynapenia is the age-related loss of SMS, usually identified as a low HGS in older adults (25). The simultaneous presence of dynapenia and obesity in older individuals has led to recognition of a more comprehensive complex metabolic syndrome known as dynapenic obesity (26). Clinically, its diagnosis is associated with numerous adverse physical and mental health outcomes (27, 28). Low HGS is also a key component of sarcopenia, a geriatric syndrome characterized by progressive, age-related loss of SMS and SMM or physical performance. Severity of sarcopenia is confirmed by findings of low SMM and physical performance, respectively (29). The simultaneous occurrence of sarcopenia and obesity is referred to as sarcopenic obesity, which is associated with poorer health outcomes, including a higher mortality rate (30).

The findings from this study demonstrated that demographic, anthropometric, serum biochemical, and dental variables significantly explained the SMS variability in obese adults. Female sex emerged as a primary positive predictor of low SMS, a result consistently reported in the literature. This difference is attributable to variations in body composition, with men typically exhibiting greater SMS (31, 32). In the study by Pérez et al. (33), men demonstrated higher HGS in both absolute and relative terms, with women presenting approximately 52%–62% of the strength observed in men. Hormonal factors also play an essential role in these differences between the sexes. Sartorio et al. (34) suggested that sex hormones, particularly testosterone, further contribute to the development of SMM, leading to greater SMS in men. During adolescence, men's muscle mass increases is more pronounced, while women tend to accumulate more white adipose tissue. Additionally, even after adjusting for body mass, SMS remains lower in women, suggesting that muscle mass distribution and neuromuscular characteristics also contribute to these differences (35).

The pathogenesis of muscle weakness in individuals with obesity remains unclear. In healthy adults, skeletal muscle tissue plays a vital role in endocrine metabolic regulation (36). During the life course, SMM and SMS gradually decline while redistribution of white adipose tissue increases, a process mediated by endocrine, metabolic, and neural changes associated with aging. However, habits such as physical activity and diet may accelerate these changes in SMM and white adipose tissue (37). Notably, obesity impairs muscle mass and strength, with potential driving factors such as increased oxidative stress, persistent systemic inflammation, anabolic resistance, a predominance of type I muscle fibers, and greater intramyocellular lipid accumulation (6). Individuals with higher BMI may exhibit a greater SMM but decreased SMS (38).

Among the anthropometric and body composition variables with predictive capacity for low SMS in obese adults, lower height, increased body weight, and lower SMM were significantly associated with reduced SMS. Height functions as a structural factor, often relating to limb length and biomechanical leverage. Lower SMM emerged as a predictor of low SMS, reflecting diminished functional capacity. These findings corroborate previous studies that highlight the importance of lean mass in maintaining muscular strength and physical function (39, 40). In contrast, total body weight exhibited a negative association with lower SMS, potentially due to the adverse effects of adiposity on neuromuscular efficiency and the contribution of inflammatory and metabolic pathways that impair skeletal muscle contractility (6, 41, 42). Although total body weight is correlated with body volume, its association with strength is mediated by the balance between lean and fat mass (43, 44). Newman et al. (45) reported that FM is negatively associated with multiple domains of physical performance, compromising mobility and functional capacity.

Conversely, while lean mass is important, its effect on performance is more substantial when assessed relative to FM. These results suggest that excessive body weight is not the sole factor limiting functionality; rather, the predominance of metabolically inactive tissues, such as adipose tissue, is critical. A recent study highlighted that elevated FM can attenuate the positive relationship between muscle mass and functional performance (46). Increased adiposity places additional mechanical load on the musculoskeletal system, impeding functional tasks and promoting a greater risk of falls and disabilities, especially in the elderly and obese. Consequently, physical functionality assessment should account for both total body weight and the distribution of lean and fat mass, guiding more effective interventions for improving functional performance.

High-sensitivity C-reactive protein, a biomarker of systemic inflammation, was also negatively associated with HGS, reinforcing the hypothesis that chronic subclinical inflammation compromises muscle function.

In terms of oral health status, greater NMT and gingival bleeding predicted low SMS in obese adults. These findings support the hypothesis that compromised oral conditions, especially those associated with chronic inflammation such as periodontitis, may exert deleterious systemic consequences. Persistent oral inflammation may increase systemic inflammatory burden, negatively affecting muscle metabolism and body composition (47, 48). Furthermore, tooth loss can reduce masticatory efficiency, leading to diets with lower nutrient density that subsequently impair muscle mass and functionality (12, 49).

Despite the promise shown by our findings, this study has certain limitations. Cross-sectional design prevents us from establishing cause-and-effect relationships. Additionally, the sample size may have limited the statistical power and the ability to detect significant associations through more robust multivariate analyses. Furthermore, specific characteristics of the study population may restrict the generalizability of these results. Therefore, the associations observed here should be confirmed in future studies with larger samples. Anthropometric and BIA assessments of SMM in obese adults are less accurate than gold-standard imaging methods (computed tomography and magnetic resonance) and even the dual-energy x-ray absorptiometry, for body composition assessments, which are associated with high financial costs for acquisition and maintenance and are in high demand in public health systems. Our future research will aim to explore correlations between anthropometric measurements and lifestyle factors, including diet, physical activity, and exercise, in obese adults. It is also essential to investigate the role of circulating inflammatory and metabolic biomarkers from blood and saliva in the incidence and progression of skeletal muscle weakness, considering the accumulation of body fat from childhood through old age.

The present findings underscore the importance of a multidimensional approach in health care for obese adults, emphasizing the importance of oral health professionals in screening for low SMS. The predictive diagnostic model supports risk stratification, facilitates prevention of early functional decline, and informs health promotion strategies in public healthcare settings. Further longitudinal studies are recommended to validate and refine the model in different populations and to evaluate the effectiveness of interventions targeting identified factors on handgrip strength and clinical outcomes related to functionality, quality of life, morbidity, and mortality.

## 5 Conclusion

In conclusion, low SMS is prevalent among obese adults. Predictors include female sex, lower height, increased body weight, and SMM estimative, gingival bleeding, and greater NMT parameters. Public primary healthcare services should prioritize its diagnosis in adults who are at increased risk for sarcopenic obesity, using reliable, reproducible, economical, and easily administered anthropometric assessments. Early detection and monitoring may contribute to developing specific preventive and therapeutic interventions.

## Data Availability

The raw data supporting the conclusions of this article will be made available by the authors, without undue reservation.
